# From Isles of Königsberg to Islets of Langerhans: Examining the Function of the Endocrine Pancreas Through Network Science

**DOI:** 10.3389/fendo.2022.922640

**Published:** 2022-06-15

**Authors:** Andraž Stožer, Marko Šterk, Eva Paradiž Leitgeb, Rene Markovič, Maša Skelin Klemen, Cara E. Ellis, Lidija Križančić Bombek, Jurij Dolenšek, Patrick E. MacDonald, Marko Gosak

**Affiliations:** ^1^ Institute of Physiology, Faculty of Medicine, University of Maribor, Maribor, Slovenia; ^2^ Department of Physics, Faculty of Natural Sciences and Mathematics, University of Maribor, Maribor, Slovenia; ^3^ Institute of Mathematics and Physics, Faculty of Electrical Engineering and Computer Science, University of Maribor, Maribor, Slovenia; ^4^ Department of Pharmacology and Alberta Diabetes Institute, University of Alberta, Edmonton, AB, Canada

**Keywords:** pancreatic islets, beta cells, calcium imaging, intercellular communication, functional networks, multilayer networks

## Abstract

Islets of Langerhans are multicellular microorgans located in the pancreas that play a central role in whole-body energy homeostasis. Through secretion of insulin and other hormones they regulate postprandial storage and interprandial usage of energy-rich nutrients. In these clusters of hormone-secreting endocrine cells, intricate cell-cell communication is essential for proper function. Electrical coupling between the insulin-secreting beta cells through gap junctions composed of connexin36 is particularly important, as it provides the required, most important, basis for coordinated responses of the beta cell population. The increasing evidence that gap-junctional communication and its modulation are vital to well-regulated secretion of insulin has stimulated immense interest in how subpopulations of heterogeneous beta cells are functionally arranged throughout the islets and how they mediate intercellular signals. In the last decade, several novel techniques have been proposed to assess cooperation between cells in islets, including the prosperous combination of multicellular imaging and network science. In the present contribution, we review recent advances related to the application of complex network approaches to uncover the functional connectivity patterns among cells within the islets. We first provide an accessible introduction to the basic principles of network theory, enumerating the measures characterizing the intercellular interactions and quantifying the functional integration and segregation of a multicellular system. Then we describe methodological approaches to construct functional beta cell networks, point out possible pitfalls, and specify the functional implications of beta cell network examinations. We continue by highlighting the recent findings obtained through advanced multicellular imaging techniques supported by network-based analyses, giving special emphasis to the current developments in both mouse and human islets, as well as outlining challenges offered by the multilayer network formalism in exploring the collective activity of islet cell populations. Finally, we emphasize that the combination of these imaging techniques and network-based analyses does not only represent an innovative concept that can be used to describe and interpret the physiology of islets, but also provides fertile ground for delineating normal from pathological function and for quantifying the changes in islet communication networks associated with the development of diabetes mellitus.

## Introduction

### From Isles of Königsberg to Network Science

**Figure 1:**
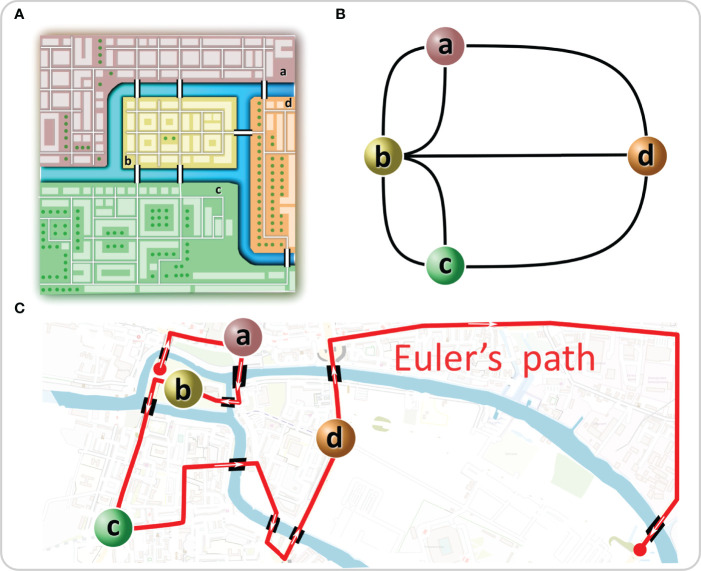
The “Königsberg bridge problem” as a graph. **(A)** In Euler’s time, one part of Königsberg (a) was connected with two bridges to the smaller isle (b) in the Pregel River and with one bridge to the larger isle (d). The same was true for the other part of the city (c). The problem was to create a path that would cross each bridge only once. **(B)** Euler solved the problem by representing the system as a graph in which the isles were represented as nodes and the bridges as links between them. **(C)** Euler´s path is shown on the current map of Kaliningrad.

Graph theory has its roots in the 18^th^ century. In 1735, Leonhard Euler became interested in a then popular brainteaser of Königsberg, today’s Kaliningrad. Kaliningrad´s center was built on four land masses, two isles on the river Pregel and two riverbanks. At that time, they were connected by seven bridges (**Figure 1A**). The problem was to cross all seven bridges in a continuous stroll, crossing each bridge exactly once. To solve the problem, Euler redrew the geographical situation of the city by constructing a graph with the land masses being represented as four nodes and the bridges being represented as seven links between them (**Figure 1B**). Euler analyzed the possibility of walking through a graph (city) using each edge (bridge) only once by considering the degree (number of edges connecting to each isle) of the vertices (isles). A path in a graph, which contains each edge only once, is called an Euler’s path. He realized that for such a walk to be feasible, there must be none or exactly two nodes with an odd number of links attached to them since any walker must both enter and leave all nodes, except the ones where the walk begins and ends^1^. All four nodes possessed an odd number of links; thus, the proposed walk was impossible (1). Interestingly, due to bombing, reconstruction, and construction of new bridges, today, there are altogether 9 bridges, one node has 3 links, two have 4 links, and the fourth has 7 links (**Figure 1C**). Therefore, a modern walk is possible in theory, but it must start on one and end on the other isle, making it hardly feasible in practice (**Figure 1C**). By solving the brainteaser in this way, Euler laid the foundation of graph theory and showed that functional implications of a particular structure can be understood and even predicted without working out all particularities.

Since its beginning, graph theory has occupied a place at the ‘pure’ end of pure mathematics. However, during the last century, analyses of social networks started gaining prominence apart from the developments in the field of mathematical graph theory (2, 3). These advances, along with a data deluge at the turn of the 21st century, have led to the birth of a new interdisciplinary field that has come to be known as network science (4, 5). This inherently multidisciplinary new discipline branch of study is now established as a backbone for describing various natural, social, and technological systems. It has catalyzed one of the most striking discoveries in complex systems research, i.e., that interactions of real-world networks follow some universal features. In a seminal paper (6), Watts and Strogatz pointed out the omnipresence of small world connectivity patterns that are highly clustered and exhibit small characteristic path lengths. Such topological structures were identified in electric power grids, neuronal and brain networks, protein-protein interactions, metabolic pathways, transportation, social networks, and food webs, to name only a few (7–10). The next decisive breakthrough was the growth and preferential attachment model that describes the universal scaling in degree distributions by Barabási and Albert (11), along with the notion that highly heterogeneous and scale-free-like networks likewise pervade biological, social, and technological systems (12–14). It is clear that the potential of graph theory to solve real-world problems is only beginning to be realized.

### From Network Science to Islets of Langerhans

Complex networks are widely applicable because they can represent and study the relationships between individual components in virtually any discrete system. In the last two decades, along with the advances in high-throughput technologies, including increasing computational power, biometric data acquisition, imaging techniques, and bioinformatics, network concepts have become an indispensable tool in biomedical research (15–23). In biomedicine, network science allows us to analyze, integrate and interpret several types of data, as well as to link them together in novel ways. Therefore, it complements the reductionist approach of systems biology, which focuses on identifying the key elements and their role in isolation. In addition to studies of gene-gene interactions (24), network science is also being used to analyze interactions between individual molecules, proteins (25, 26), signaling pathways, and more recently, to study the underlying pathophysiology of various disease processes (27–29), both at the level of the individual organs and at the systemic level. Network analyses nowadays allow the study of complex signaling pathways in health and disease, with the integrative concept being especially valuable in complex diseases with different comorbidities and multiple phenotypes (30). By understanding the bigger picture, it allows, on the one hand, the detection of potential drug targets, while at the same time offering the possibility of a systems approach to diagnosis and therapy (21, 27, 31, 32). Furthermore, under the framework of the recently emerging field of network physiology, network approaches have proven valuable to describe the structural organization and functional interrelations between different physiological and organ systems on the whole-organism level (18, 22, 33–35). Noteworthy, in the last decade perhaps the most remarkable breakthroughs of complex network analysis were achieved in neuronal physiology, where these approaches are used to quantify the brain’s functional and anatomical organization and have reached the maturity to be translated into clinical practice (36–39).

Of most importance for our present review is the fact that in recent years, network theory has been used to assess intercellular interactions in multicellular systems where nodes represent individual cells, and their locations correspond to physical positions of cells in the tissue. Functional connections between cells are created based on the temporal similarity of the measured cellular dynamics, as evaluated through the calculation of the correlation coefficient or other metrics for statistical similarity of time series. Multicellular recordings of membrane potential or intracellular calcium concentration are typically used as input signals. Network principles have thus been used to elucidate the connectivity patterns in neurons (40–44), astrocytes (45), pituitary endocrine cells (46, 47), lens epithelium cells (48, 49), hepatocytes (50), mammary epithelium cells (51), endothelial cells (52), and pancreatic acinar cells (53). Network analyses were introduced to the islet community a decade ago and are increasingly seen as a valuable tool to examine and quantify collective activity patterns in the pancreatic islets of Langerhans (18, 54–69). In these microorgans, communication among a variety of cells with unique functions and characteristics must occur to ensure proper control of metabolic homeostasis (70–73). The most prevalent cell type are the insulin-secreting beta cells, which are electrically coupled through gap junctions composed of connexin36 (Cx36) to ensure mediation of intercellular signals (74, 75). Because beta cells are intrinsically highly heterogeneous and operate in a continuously changing environment, they exhibit complex yet coherent activity patterns, that are essential to tightly controlled insulin secretion. Inspired by and contributing to the increasing awareness that both synchronized islet activity and cell-to-cell communication are altered during the pathogenesis of diabetes (76–80), tools from the armamentarium of the complex network theory are now recognized as a powerful computational framework to assess the multicellular activity in islets and to study the progression of islet dysfunction in diabetes. Therefore, we review the recent advances related to the application of network theory to study islet physiology. We first provide a comprehensible introduction to the basic principles of network theory to introduce physiologists to this rather new theoretical paradigm. Then, we demonstrate how the complex network approaches can be used to uncover the functional connectivity patterns among cells within the islets and specify their functional implications. We conclude by pointing out some challenges and possible directions for future investigation of islets and the multicellular dynamics of endocrine cells in general.

## Types of Networks and Network Metrics

The birth of modern network science began with the objective of characterizing the topology of real-world systems consisting of many interacting units. In its purest form, a graph or a network is an abstract mathematical object consisting of nodes (or vertices) which are connected by links (or edges or connections). Because of this rather simple definition, complex networks represent a general and very useful framework to describe a large variety of social (3, 8, 81), biological (17, 18, 82–85), and technological systems (86–88). Nodes and edges can be regarded as a manifestation of some properties of a system and the corresponding network is a simplified mathematical representation of the relationships (edges) between variables (nodes), that does not necessarily encompass all the details of the underlying system. For example, in online social platforms, people (nodes) are connected through friendships (links); studying these social networks can allow the study of how information spreads without knowing details about each specific person in the network. Similarly, protein-protein interaction networks, where nodes in networks represent proteins and links represent interactions between them, can lead to a systems level understanding of the cell. Furthermore, data abstraction in networks facilitates the identification of general topological characteristics in interaction patterns. The observation of small-world characteristics, universal scaling, and heterogeneity in the degree distributions, as well as the existence of community structure, are the most important features observed in a plethora of empirical networks (6, 11, 89–91).

**Figure 2:**
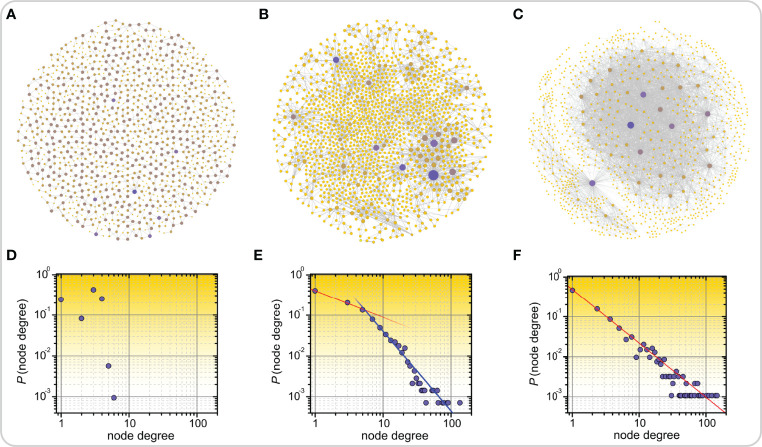
Examples of real-world networks along with their corresponding degree distributions. Road network in California **(A)**, disease-disease network in which nodes represent inherited, developmental, or acquired diseases and links represent associations between them **(B)**, and air transport network in the US **(C)**. In panels **(A-C)** highly connected nodes, i.e., hubs, are colored purple, all other nodes are colored yellow. The lower panels feature the corresponding degree distributions of networks which are categorized as single-scale **(D)**, broad-scale or weakly scale-free **(E)**, and scale-free **(F)**.

Small-world networks can be divided into three structural classes: (i) single-scale networks, (ii) broad-scale networks, and (iii) scale-free networks (8). This classification relies on the computation of the degree distribution, meaning the proportion of nodes that have a certain degree or number of edges (8, 92). **Figure 2** shows three different types of networks along with their degree distributions that were obtained from the SNAP database (93). **Figure 2A** represents the road network in California, which is a homogeneous single-scale network that is characterized by a degree distribution with a fast decay, i.e., there are no nodes with a very high degree, meaning there are no cities or towns with extremely high numbers of roads connecting to them. **Figures 2B, C** represent a disease network and the air transport network in the USA, respectively. Both of these networks are heterogeneous, as evidenced by the heavy-tailed degree distributions, indicating thereby that there are a few nodes with a very high number of connections, i.e., hub nodes. However, only the air transportation network exhibits a scale-free structure with a clear power-law degree distribution. In contrast, despite being heterogeneous, the disease network structure deviates from the pure power-law behavior. Therefore, it can be categorized as a broad-scale or a weakly scale-free network. Notably, such structures are much more common in real-world networks than rigorous scale-free networks. More specifically, due to different constraints and finite sizes, scale freeness of networks is not a ubiquitous phenomenon and therefore the term scale free network is in the literature sometimes loosely applied to different types of heterogeneous networks with heavy-tailed degree distributions (14). Nevertheless, from the functional point of view, when dealing with real-life networks, it is much more important to know whether a network´s degree distribution is heavy-tailed, so that we are aware of the existence of hub nodes, than whether it exactly follows a power-law (94, 95).

**Figure 3:**
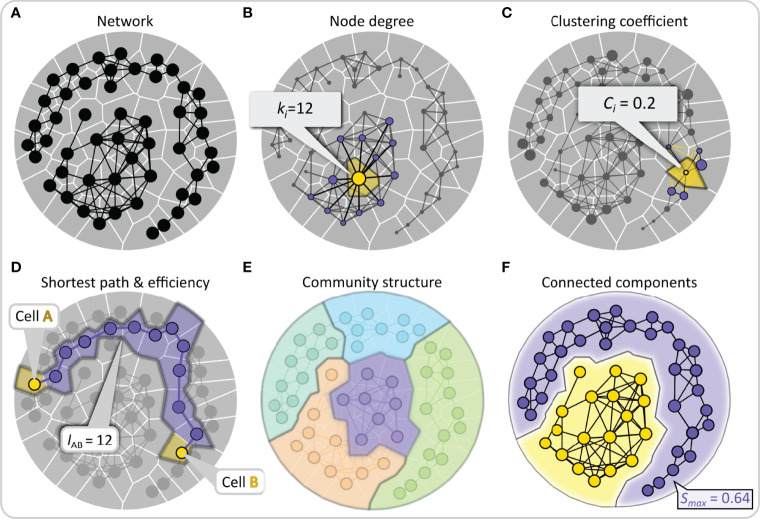
Quantifying intercellular connectivity patterns with conventional network metrics. **(A)** Schematic representation of a multicellular system as a network composed of nodes (cells) and edges (connections or functional associations between cells). **(B)** Nodes are scaled based on the number of the neighbors to which they are directly connected, i.e., their degree. This feature is shown schematically for the yellow node, with the direct neighbors colored purple. **(C)** The clustering coefficient describes the tendency of nodes to cluster together and is defined as the number of existing connections between the neighbors of that node divided by the number of all possible connections between them. This property is illustrated by the example of the yellow-colored node, to which non-existing yellow edges between the purple neighboring nodes have been added. **(D)** The shortest path length between any two nodes is the number of edges that form the shortest path between them. Lower average shortest paths of a network indicate more efficient communication abilities. This feature is highlighted by two yellow nodes representing the source and destination nodes and the purple-colored nodes forming the shortest path. **(E)** A community consists of a group of nodes that have a higher connection density compared to the whole network. A well-pronounced community structure implies an internal network organization into functional modules. In panel **(E)**, nodes are color-coded according to their membership in a community. **(F)** A group of nodes that are directly or indirectly connected to each other forms a connected component and stands for reachability within the network. The relative largest connected component representing the fraction of nodes that are connected either directly or indirectly is denoted by *S*_max_.

The most commonly used network metrics applicable to studying multicellular networks are schematically presented in **Figure 3** (for more details see (18, 96)). By simply counting the number of links of a node, we define its degree *k_i_*, and by averaging the degrees of all nodes, we can calculate the average degree of the network. The degree of a node is an indicator of its importance. By drawing a degree distribution, we can then distinguish between different types of networks, as already outlined above. To estimate whether nodes in the network are connected in a more segregated or a more integrated way, we can calculate the so-called local clustering coefficient *C_i_*, which is a measure of how well adjacent nodes are interconnected, while the average clustering coefficient is defined as the average of all local clustering coefficients and indicates the overall interconnectedness. From a functional point of view, high clustering imparts resilience to removal of nodes since a signal will reach all neighbors of a removed cell if they are connected with each other. A metric for evaluating the quality of integration is the global efficiency of the networks, which is defined as the inverse sum of all shortest path lengths between all accessible pairs of nodes. Higher values of global efficiency (i.e., shorter average path lengths) indicate better communication capability between nodes. Importantly, the trade-off between clustering coefficient and the global efficiency is also used to measure the degree of small-worldness in the network, small-world networks are expected to simultaneously display both high integration and segregation, i.e., high global efficiency and clustering (97). Moreover, it is sometimes important to find communities or subsets of nodes that are densely connected, and the extent to which the network is divided into communities can also be evaluated using the so-called modularity metric. A well-pronounced community structure in a network indicates a functional specialization of specific regions or subnetworks. In islets of Langerhans, this could indicate presence of islets within islets or islets that merged during ontogenesis. Lastly, network analysis can be used to identify connected components, defined as a subset of nodes that can reach any other node by traversing links with the goal of describing the reachability of the network structure and finding isolated subnetworks. Of particular importance is the network’s largest component *S*_max_, i.e., the maximal set of mutually connected nodes, which reflects the degree of integration and resilience of the network. All network measures described here and in **Figure 3** can easily be applied to intercellular interaction patterns within the pancreatic islets once multicellular networks are established from experimental data.

## Designing Functional Beta Cell Networks

Networks are a convenient abstraction tool for exploring how relationships and interactions between individual components give rise to emergent dynamics. When mapping cellular associations, an important distinction must be drawn between how cells are physically connected and how the actual information transfer occurs, affecting the collective activity of cellular populations. Formally, we distinguish between structural and functional networks, even though it is known that the structure and function in networks are closely intertwined (98). Structural intercellular networks describe the patterns of cellular morphology arrangement and provide the mechanistic substrate for intercellular signal transfer. Structural network analysis has already been utilized to study the cytoarchitecture of the islets. Specifically, network-based methods have been used to assess the spatial organization of cells and their homo- and hetero-typic contacts (99–104), to elucidate the principles of beta cell arrangement in normal and diabetic islets (105), to infer the structural basis for paracrine regulation of delta cells (106), and to study the arrangement of endocrine cells regarding the vascular network in the islets (107). While structural network analysis represents a vibrant topic in the islet community, it is beyond the scope of this review.

**Figure 4:**
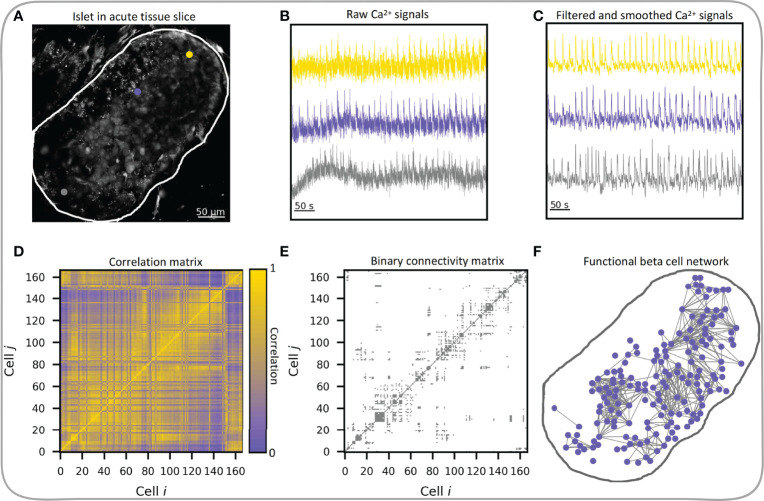
Workflow of functional beta cell network extraction. **(A)** Confocal image of islet of Langerhans in acute tissue slice with three indicated beta cells (gray, yellow and violet dots) and indicated islet outline (white curve), exported raw Ca^2+^ signals of the three beta cells **(B)** and processed Ca^2+^ signals **(C)** of the same three cells. **(D)** Pearson correlation coefficient matrix based on filtered and smoothed Ca^2+^ signals of all cell pairs in the islet. **(E)** Extracted binary connectivity matrix based on the thresholded correlation matrix; only cell pairs whose correlations exceeded *R*_th_ = 0.82 were considered connected. **(F)** The corresponding functional beta cell network. Blue dots denote individual beta cells and gray lines represent functional connections between highly synchronized cells.

Here, we focus exclusively on functional multicellular networks, which derive from the system’s dynamics. In functional connectivity maps, nodes represent individual beta cells and connections between them are established based on the temporal similarity of the measured cellular dynamics, as typically assessed by statistical similarity of calcium signals (18). The creation of functional beta cell networks has become feasible with the development of functional multicellular calcium imaging techniques. Two earliest adopters of these techniques in conjunction with network-based analysis were Hodson et al. (54) in isolated islets and our group in tissue slices (62), where we represented cells as network nodes and functional connections between cells as network edges. In both studies as well as in most studies that followed, a thresholding of the correlation matrix was used to construct functional networks, where two cells were deemed functionally connected if their correlation coefficient exceeded a preset threshold level. The procedure is schematically presented in **Figure 4** and encompasses the processing of the recorded Ca^2+^ time series. In most of our previous works, we focused exclusively on the fast Ca^2+^ oscillations that propagate across the islets in the form of intercellular waves. To this aim, the signals were band-pass filtered and smoothed to remove noise and variations in basal Ca^2+^ levels. We will discuss how preprocessing of recorded traces affects the network characteristics in the next chapter.

The methodology to create such functional connectivity maps originates from neuroscience, where similar approaches have been used to study functional associations between brain regions or neurons (36, 108). However, a drawback of the method is that it relies on a somewhat arbitrary connectivity threshold selection (109–112). As one of the criteria it was suggested that the selected threshold value should be chosen at *R*>0.7, such that more than half of variance is explained by the correlation (*R* ^2^>0.49). Alternatively, one can fix the average node degree, such that it reflects a physiologically relevant value (i.e., the expected number of connections in the observed tissue). By this means, the correlation threshold value for intercellular connectivity is iteratively adjusted until the resulting network has the desired edge density. In this context, the average node degree itself no longer has any descriptive meaning when comparing different networks, however, the underlying network parameters and degree distributions can differ greatly. If, for example, individual nodes of a network are poorly correlated, the resulting network will have more random-network-like attributes (high efficiency, very low clustering, high modularity), compared to highly correlated nodes which result in a more ordered network structure (high clustering, lower efficiency, and lower modularity). Therefore, this methodology should not be used when the signals in different islets differ substantially. As an alternative, the minimum spanning tree network can be used. This method represents an unbiased network construction method which relies on the fully connected, weighted and undirected graph of the system and extracts only the sub-network which contains *N*-1 strongest connections (*N* – total number of nodes) with no disconnected nodes or circular connections. It has been, for example, successfully applied in brain research (113) and in assessing the collective dynamics of cryptocurrencies (114) or energy consumption profiles (115). The lack of disconnected nodes and the simple, barebone representation are the main advantages of this method, since disconnected nodes can represent a problem for previously described approaches. The main drawback is the lack of circular connections, consequently zero clustering coefficient and an overall poorer description of the underlying system. Networks can also be constructed from binarized time series and in this case the so-called coactivity coefficient is used to evaluate synchronicity between cells (64, 66, 116). While this method does objectively capture the level of synchronicity between cell pairs, it should not be used when there are notable differences in the activities of individual cells, as it might lead to spurious results. Another approach, popular in neuroscience for inferring the connectivity between brain regions, is the Granger causality (117). As the name suggests, this method is not only used for determining functional connectivity between brain regions, but also for the direction (causality) of these connections and was also successfully utilized in beta cell research for identifying first responder cells in mouse islets following glucose stimulation (67). Finally, it should be noted that particularly in neuroscience more advanced methods to quantify statistical interdependencies between active nodes are gaining attention, such as the partial cross-correlation (114, 118, 119), dynamic time warping (120, 121), and minimum jump cost (122, 123).

## Topological Analysis of Functional Beta Cell Networks

Beta cells respond to stimulation with tightly coupled oscillations in membrane potential and intracellular calcium concentration [Ca^2+^]_IC_ leading to insulin secretion (124–128). At present, Ca^2+^ oscillations can be observed in two distinct time domains: i) fast Ca^2+^ oscillations under 20 s long, appearing with a frequency of about 5 min^-1^ and corresponding to beta cell bursting electrical activity (55, 124, 129, 130), and ii) the underlying slow oscillations with a frequency of around 0.1-0.2 min^-1^ (56, 131, 132). Gap junctional coupling of beta cells *via* Cx36 allows for the electrical linkage as well as the exchange of small molecules between neighboring cells and mediates principally the coherence of fast Ca^2+^ dynamics (133, 134). Correlations between slow Ca^2+^ oscillations can be driven by metabolic coupling of neighboring cells, *via* feedback onto the slow dynamics by the fast dynamics due to same electrical coupling (77, 135–139), as well as by intrinsic metabolic characteristics of beta cells (61). Since both fast and slow oscillations are synchronized among the cells in an islet, they both contribute to functional connectivity networks derived from Ca^2+^ signals (56, 133, 140, 141).

In our first study in which we employed network analysis to investigate functional connectivity we showed that beta cells are most synchronized and densely connected during the activation phase following the administration of stimulatory levels of glucose and during the deactivation phase after glucose stimulation has ceased. During these periods, the functional connections were mostly independent of the Euclidean distance between cells within the islet. This behavior may be because beta cells individually yet synchronously move from basal [Ca^2+^]_IC_ to the much higher “plateau” level required to trigger insulin secretion upon stimulation, and back to baseline upon termination of stimulation, yielding highly correlated signals. Notably, similar observations were later also made in measurements on zebrafish islets *in vivo* (67). Furthermore, in the phase of sustained activity (plateau phase), an intermediate level of synchronization was observed, and the length of functional connections was considerably lower, suggesting that the cells are predominantly synchronized *via* calcium waves which spread across the islet (62). Moreover, initial studies of beta cell networks have revealed that functional beta cell connectivity patterns are small-world networks dominated by a small subset of cells with a high degree of functional connectivity, i.e., hub cells (62, 64). The high level of heterogeneity and the presence of long-range correlations were perhaps the most striking observations of the earliest studies. The existence of hub cells was also linked with metabolic profiles, which will be addressed in more detail in section 5. The presence of long-range connections that give rise to small-world characteristics was attributed to various reasons associated with a complex multicellular dynamics, whereby heterogeneity and a heterogeneous coupling were highlighted also in theoretical models (142, 143).

**Figure 5:**
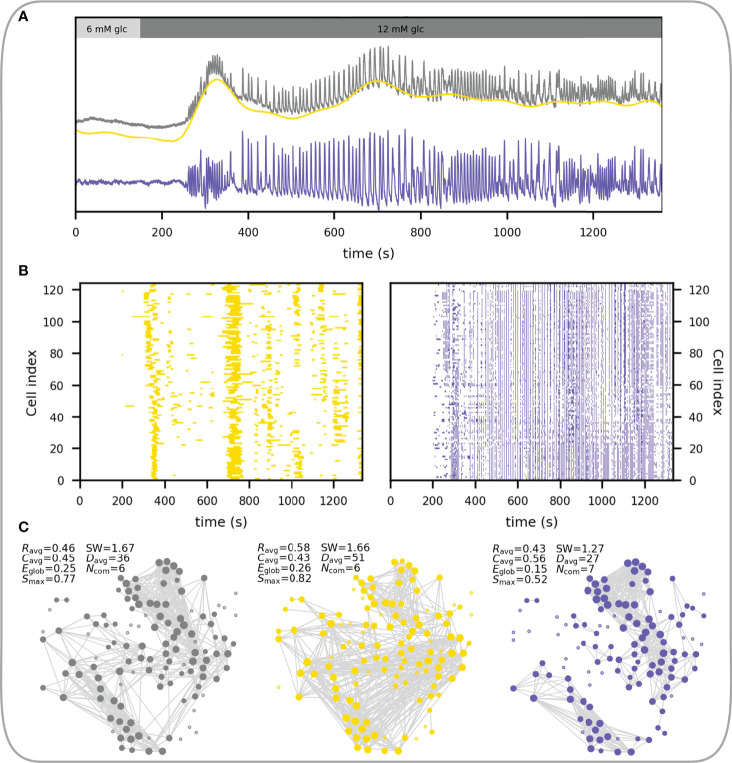
Constructing functional beta cell networks from different types of Ca^2+^ signals. **(A)** Raw average Ca^2+^ signal of a representative islet (gray line) with extracted slow (yellow line) and fast (blue line) signal components. **(B)** Raster plots showing the activity of the slow (left, yellow dots) and fast (right, blue dots) Ca^2+^ signals of all cells in the islet. **(C)** Correlation-based functional networks constructed from unprocessed (left), slow- (middle) and fast (right) Ca^2+^ signals. Networks were constructed with a fixed average network node degree *k*_avg_ = 8.3. The following parameters for each functional network are provided: average correlation coefficient (*R*_avg_), average clustering coefficient (*C*_avg_), global efficiency (*E*_glob_), largest connected component (*S*_max_), small-world coefficient (SW), average physical length of functional connections (*D*_avg_) and the number of communities (*N*_com_).

The structure of functional networks depends heavily on the type of the input signal. As explained above, the measured Ca^2+^ dynamics comprises different temporal domains (**Figure 5A**), whose activity is coordinated across the islets in a different manner. In other words, beta cells have fluctuations in intracellular calcium concentrations at both faster and slower oscillatory rates, and the networks constructed from these separate temporal domains have differing properties. **Figure 5A** shows a representative beta cell response, where the raw, unfiltered [Ca^2+^]_IC_ dynamics is shown in gray and yellow and violet traces represent the filtered signal with only the slow and only the fast oscillatory component, respectively. In **Figure 5B**, the corresponding raster plots of the binarized fast and slow activity are presented and it can be noticed that both types of oscillatory dynamics display coordination across the islet. With these different input signals, three different functional networks can be constructed and characterized (**Figure 5C**). Metabolically driven slow dynamic component gives rise to substantially longer functional connections, due to global slow Ca^2+^ waves occurring on a broader temporal scale (56). The slow component gives rise to a higher average correlation (*R*_avg_), while the fast component results in higher clustering (*C*_avg_), pointing out a denser interconnectivity between neighboring cells. Moreover, the network built upon the fast Ca^2+^ component results in a lower global efficiency (*E*_glob_) compared to both the slow and the unfiltered signal, denoting a longer characteristic path length between beta cells. Despite the higher clustering in the fast component, the lack of long-range connections leads to a decreased measure of small-worldness (SW). This indicates that the slow component importantly contributes to the level of small-worldness of the beta cell functional connectivity networks, which is an important aspect to consider when unprocessed signals are used for the analysis. Therefore, in addition to the insights gained by separating Ca^2+^ signals into various components (fast vs. slow vs. a combination of both), further methodological steps, i.e., signal filtration, affect the characteristics of functional networks and their implications in islet function. Finally, it should be emphasized that functional connectivity describes a statistical relationship between the measured signals and therefore long enough intervals should be used to obtain relevant results, particularly when slow oscillations are analyzed, which display a relatively low temporal density of events (typically on the order of magnitude of 0.1 min^-1^).

**Figure 6:**
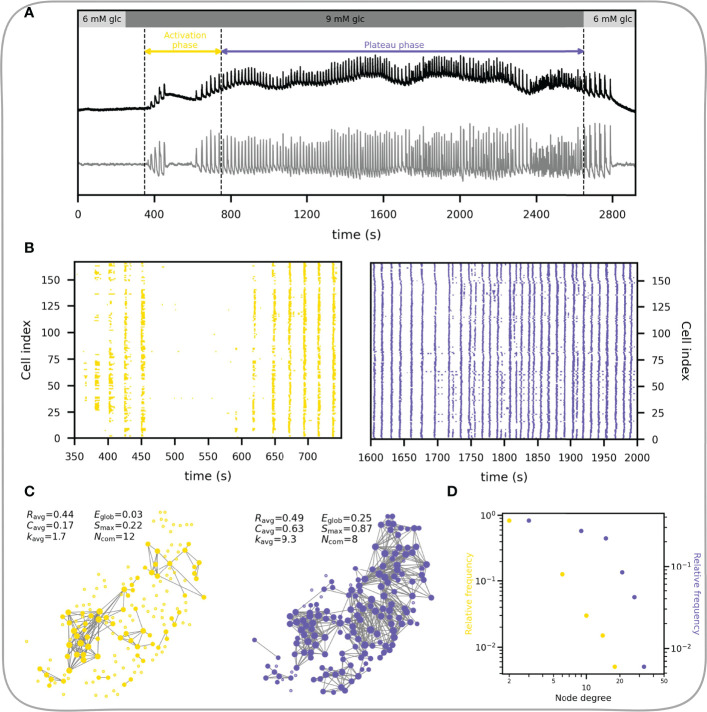
Characteristics of the functional beta cell network in response to stimulation. **(A)** Average Ca^2+^ signal of an islet subjected to a 6-9-6 mM glucose stimulation protocol. Black line represents the unprocessed Ca^2+^ signal and the gray line represents the extracted fast component of cellular activity. Colored horizontal arrows indicate the selected activation and plateau phases for further analysis. **(B)** Raster plots showing the binarized fast component of beta cell activity for the activation (left) and plateau (right) phase as indicated in panel **(A)**. **(C)** Functional beta cell networks constructed for both phases (left, activation phase; right, plateau phase) based on fast signal components of all cells with a correlation coefficient threshold for connectivity *R*_th_ = 0.8. The following parameters for each functional network are provided: average correlation coefficient (*R*_avg_), average clustering coefficient (*C*_avg_), average network node degree (*k*_avg_), global efficiency (*E*_glob_), largest connected component (*S*_max_) and the number of communities (*N*_com_). **(D)** Node degree distributions for the networks extracted in the activation (yellow dots) and plateau phase (blue dots).

In what follows we focus on the fast component of Ca^2+^ response. Beta cells respond to glucose stimulation in a biphasic manner (144–146). Following exposure to, there is an activation phase with a transient rise in [Ca^2+^]_IC_ and fast Ca^2+^ oscillations, during which beta cells are progressively recruited and begin to operate more synchronously. Heterogeneous activation of beta cell clusters during activation is reflected in local Ca^2+^ waves that are heterogeneous in size (55, 145, 147). Activation is then followed by a stable plateau phase with synchronized, regular oscillations and islet-wide activation of beta cells, as indicated with the replacement of local Ca^2+^ waves with global Ca^2+^ waves (55, 147). This behavior is demonstrated in **Figures 6A, B**. The corresponding temporal evolution of functional networks throughout stimulation is reflected in its characteristics (**Figure 6C**), with a higher average node degree, clustering coefficient, largest component, and a smaller number of communities during the plateau phase. In other words, with the increase in activity and coordination cells become more connected, the network becomes denser and more integrated, with fewer and bigger communities.

The node degree distribution during the plateau phase decreases roughly potentially with a cut-off in the tail, i.e., following an exponentially truncated power law signifying the broad-scale nature of functional networks (**Figure 6D**) (55, 62). We wish to point out that physical limitations prevent the emergence of truly scale-free networks, i.e., a cell cannot be functionally connected to more cells than there are in a tissue, and consequently, they are not as ubiquitous in real world as is often presumed (14). It is worth emphasizing that the noted heterogeneity in the degree distribution implies the existence of highly connected beta cells, i.e., hub cells that are in this islet connected to more than 20% of other cells. While some studies have demonstrated a particular importance of this highly connected subpopulation of beta cells in orchestrating collective beta cell activity in healthy islets (148), as well as their crucial role in pathogenesis of diabetes (54, 58, 64), the evidence is not unanimous and many details about their characteristics and the part they play in islet coordination remain to be elucidated (55, 149–151).

## Functional Implications of Beta Cell Network Analysis

### From Classical Physiological Parameters to Functional Connectivity

Collective activity of beta cells achieved through tightly regulated electrical coupling of neighboring beta cells (and perhaps also other cell types) *via* Cx36 is vital to well-regulated insulin secretion (152). Functional multicellular imaging has allowed deeper insight into the importance of multicellular cooperation and functional connectivity of beta cells within an islet (73, 153–155). The response of islet**s** to glucose depends on the level of stimulation. With increasing glucose concentration from substimulatory (< 6 mM) to supraphysiological glucose concentrations (> 10mM), the delays to activation of cells shorten (advancement), and the fraction of beta cell population involved in the response increases (recruitment) (55, 145, 156–159). Also, the delay between the activation of cells that respond first (called first responders) and beta cells in the same islet that activate later is shortened (55, 156, 160). Compared with dissociated or uncoupled cells, the probability density of cell activation in coupled beta cells in islets is narrower and thus the dose response curve is much steeper. Importantly, all of these features of the activation phase agree with model predictions, as excellently reviewed recently by Peercy and Sherman (60). In the plateau phase, the glucose dependency or the dose response is evident as an increase in active time or so-called duty cycle, which is in good agreement with insulin secretion (55, 160–164). During deactivation, the delay before cells turn off is longer for higher preceding glucose stimulation, however, the heterogeneity between cells is less than during activation (55, 156, 160). Importantly, a coordinated cessation of activity is important, as it prevents hypoglycemia. Data suggest that this last phase of response may be disrupted in uncoupled beta cells and under diabetogenic conditions (66, 152). Looking at the functional connectivity of beta cells, a clear transition from more segregated to more integrated networks can be observed with increasing glucose concentrations (55, 63). These changes in activity and functional connectivity are accompanied by a switch from more local to more global [Ca^2+^]_IC_ waves (58, 147). However, despite the highly integrated functional connectivity in high glucose, an islet cannot be regarded as a uniform supercell sharing identical activity in all its parts. Rather, based on differences in activation times, activity during the plateau phase, the origin of [Ca^2+^]_IC_ waves, and the number of connections in functional networks, distinct beta cell subpopulations presumed to have distinct impacts on islet activity have been defined (54, 55, 59, 62, 64, 67, 165).

### Beta Cell Subpopulations and Functional Heterogeneity

**Figure 7:**
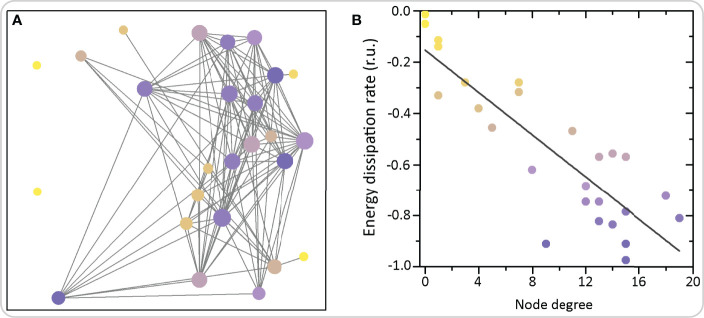
Functional network architecture of beta cells and the corresponding energy dissipation rates. **(A)** Each circle represents the physical position of a cell inside the islet and the connections signify functional connections. Colors of circles denote the average dissipation rates calculated as the sum of Lyapunov exponents (see Ref (167). for further details). **(B)** The average dissipation rate reflecting the rate of energy consumption of individual cells as a function of the node degree, i.e., number of functional connections. The grey line denotes the linearly decreasing trend showing that highly connected nodes exhibit higher dissipation and energy consumption rates.

Beta cells with a high number of links in functional networks have been termed hub cells. They were suggested to govern the functioning of islet networks by initiating [Ca^2+^]_IC_ waves (64, 67). According to this view, non-hub cells are believed to only follow metabolic, electrical, and other signaling cues from the hub cells (166). Advanced optogenetic and photopharmacological investigations indicated that hub cells are metabolically highly active, exhibit hyperpolarized mitochondria and have a lower insulin content, thus resembling a transcriptionally immature phenotype due to the low expression levels of signature beta cell transcription factors. Notably, energetic demands of the functionally most connected cells can also be estimated by analyzing details of [Ca^2+^]_IC_ dynamics. More specifically, we employed methods of nonlinear time series analysis to reconstruct the phase space and to compute the dissipative characteristics of individual cells within the network (167). Hub cells exhibit the highest energy dissipation rates, which was also predicted theoretically. This concept is demonstrated in **Figure 7**, where we show an exemplary islet with color-coded energy dissipation rates of individual beta cells. In addition, the coordinated collective response of beta cells to elevated glucose was blunted after silencing the hubs, and restored after specific stimulation, thus suggesting that they may be necessary for coordinated islet [Ca^2+^]_IC_ dynamics (64, 67). At present, these findings cannot be completely reconciled with electrophysiological and modelling findings, for reasons that arguably originate in different methodological approaches [for a recent review see (60)]. Our current understanding suggests that in a system of coupled cells of which all are intrinsically able to oscillate, silencing a small proportion of cells with the highest number of functional connections cannot prevent or importantly change the activity of the remaining cells, except close to the threshold for stimulation where the majority of cells would otherwise not be active by themselves (60, 143, 148, 149). Further experiments in a near-threshold glucose milieu with ablation of hub cells rather than their silencing may further aid to our understanding of the role of hub cells in the islet syncytium. Importantly, similar strategies could be used in future studies to assess the importance of other subpopulations of cells discussed below.

Second, a large degree of beta cell heterogeneity is also evident from coordinated [Ca^2+^]_IC_ waves that propagate across islets and seem to consistently emerge from specific subpopulations of beta cells named wave initiators or pacemakers. These cells could correspond to highly glucose responsive beta cells that have increased glucokinase activity (165, 168, 169). Interestingly, depending on the model employed, they can also exhibit a lower NAD(P)H response and a faster natural oscillation frequency than other cells in the islet, including hub cells. Therefore, they are more likely to depolarize first in response to stimulating glucose concentrations and send depolarizing currents to neighboring cells (165, 170). At present, it is undisputable that the great majority of [Ca^2+^]_IC_ waves are initiated in a limited number of cells or regions, which do not necessarily overlap with the regions with most functional connections (58, 170). Also, it remains to be shown, whether their removal critically impacts the islet as a whole and which mechanistic substrates make them initiate the waves.

Third, analyzing with single cell resolution also the activation phase enables a comparison of behavior during this phase with the one on the plateau. Recent data suggest that the so-called leaders or first responders tend to cluster in groups that become larger in higher glucose and that tend to be more active, i.e., have longer active times, also during the plateau phase (55). Their removal seems to be able to delay the onset and diminish the amplitude of subsequent oscillations (67), but they are dispensable in the sense that if they are ablated another cell will become a first responder (68). Similarly, in human islets, during the first phase of glucose response, small clusters of beta cells with high activity govern the response, whereas during the second phase, the electrical coupling becomes more important to synchronize large multicellular functional clusters (146). Moreover, from the network point of view, cells with the most functional connections tend to activate sooner (55), but not necessarily be the first responders (68). Interestingly, the ability of cells to activate first in response to glucose seems to depend less on glucokinase activity than on the resting K_ATP_ and junctional conductance, which may in addition to intra-islet differences between cells (60, 68) also explain some inter-strain differences in activation (152, 160). At present, there is not enough consistent evidence for a significant overlap between hubs, first responders, and wave initiators. However, one feature which is consistently present in both experimental and modeling studies in hub cells is their higher-than-average active time or duty cycle. This may be due to the fact that the hub cells participate in the great majority of [Ca^2+^]_IC_ waves and that their [Ca^2+^]_IC_ oscillations may be a bit longer and perhaps more stable than the ones in other cells, which is also able to explain why their signals are similar to signals from many other cells and thus their functional connectivity is high (55, 58, 60, 68, 170).

Finally, functional differences between beta cell subpopulations may change with time and experimental conditions. For instance, heretofore, the described functional roles have not been tracked over long periods of time and are thus not necessarily stable properties. Additionally, the cells that are first responders to glucose may not necessarily be first responders to some other secretagogue, and the same holds true for the role of pacemakers and hubs. Moreover, it is reasonable to assume that to some extent some traits may be shared between different subpopulations, e.g., some of the first responders to glucose may be among the first responders to GLP-1 or among the hub cells. This may again depend on the experimenter’s definition of the size of subpopulation, with larger subpopulations possibly generating more overlap than more narrowly defined subpopulations. In other words, some of the first responders will with large probability be among the hubs if one sixth or even one third of the fastest responding cells and cells with the highest degree are defined as first responders and hubs, respectively, compared with a lower probability for overlap if the cut-off is set at one tenth. On the other hand, the overlap may have to do with genuine biological properties of beta cells. In the case of different or the same cells being first responders for different secretagogues, a cell´s role as a first responder may be determined by the relative importance of a given component in the stimulus-secretion coupling cascade in this cell. In addition to resolving the controversies regarding the importance of specific subpopulations for normal and pathological islet functioning, future studies shall therefore also shed light on the temporal persistence of these roles and their sensitivity to experimental conditions, such as different secretagogues. Moreover, they shall pinpoint additional subpopulations of cells and dissect into more detail their molecular, structural, and additional functional signatures. Additionally, a consensus among different research groups would be welcome on the nomenclature and cut-off values for determining subpopulation sizes. For additional details on the role of subpopulations in human islets, please see *Human Islets and Coordinated Beta Cell Activity in Health and Disease*, and for some suggestions on how mathematical modelling and multilayer networks may help address the issue of different subpopulations, please see *Computational Models of Beta Cell Networks* and *Islets as Multilayer Networks* below. Finally, for more details on the role of heterogeneity and different beta cell subpopulations in islets, as well as some suggestions for future studies, we wish to refer the reader to some other recent articles addressing this topic in detail (55, 58–60).

### Altered Connectivity in Beta Cell Networks and Its Relevance in Diabetes

Adequate intercellular electrical coupling through gap junctions is essential for synchronized beta cell activity. In basal glucose, insulin release is low since the less active beta cells keep the intrinsically more active beta cells quiescent through junctional hyperpolarization or clamping (152, 171). In contrast, during increasing stimulation, more and more cells become active and intercellular coupling may facilitate recruitment of the least active cells. In Cx36 knock-out (Cx36 KO) mice, the response of beta cells to glucose resembles that of dispersed cells in culture including increased basal and lower stimulated insulin secretion, thereby confirming the theoretically predicted role of gap-junctional coupling for the coordinated collective response (60, 77, 78).

Importantly, prolonged exposure to high concentrations of glucose and fatty acids, as expected in diabetes, was found to downregulate Cx36 in mice, rats and humans, and may disrupt the pattern of intercellular synchronization (172–174). Similarly, studies on Cx36 KO mouse models have also shown an impairment of normal oscillatory patterns of insulin secretion elicited by glucose and a diabetic phenotype (77, 78, 152, 175). Moreover, a decrease in Cx36 protein and a smaller size of gap junction plaques were also observed in prediabetic C57BL/6 mice fed a high-fat-diet for 60 days (176). Similarly, in diabetic ob/ob mice the Cx36 protein level is significantly reduced despite a preserved Cx36 mRNA level, pointing to a decrease in protein synthesis and/or inappropriate gap junction organization, leading to deficient electrical coupling between cells (172). Also the synchronicity of Ca^2+^ oscillations in ob/ob mice is disturbed, disrupting the normal insulin secretion pattern (172, 177). From a mechanistic point of view, in (pre-)diabetes, a decrease in Cx36 coupling could be a consequence of increased concentrations of pro-inflammatory cytokines. Interestingly, in both mouse and human islets a cytokine-mediated decrease in coupling can be prevented with pharmacological interventions which increase intracellular cAMP. In this case, in mouse islets the glucose-stimulated calcium signaling was preserved by increasing the levels of gap junction protein Cx36 on the plasma membrane using exendin-4, a glucagon-like peptide (GLP-1) receptor agonist (178).

To protect islets from high-fat-diet induced impairment of beta cell gap junction coupling and to preserve proper Ca^2+^ signaling, including Ca^2+^ oscillation coordination and amplitude, a relatively simple intervention, namely a 40% caloric restriction, seems to be very efficient, mirroring similar beneficial effects of caloric restriction in human patients (179). Additionally, trying to decipher the effects of vertical sleeve gastrectomy (VSG) on beta cell function in obese mice, Akalestou et al. have shown that Ca^2+^ dynamics as well as the number and strength of connections between beta cells increase within 8-10 weeks post-surgery, which could be attributed to the strong influence of GLP-1 on islet functioning (57). GLP-1 in physiological picomolar concentrations augments postprandial insulin secretion. However, it significantly enhances beta cell cluster activity, coupling, and coordination only in the second phase of insulin secretion as shown using a microfluidic system with multielectrode arrays (146). As evident from the above findings, an important impact on Ca^2+^ oscillations is mediated by cAMP acting downstream through at least two different pathways, namely the guanine nucleotide exchange factor Epac2A and the protein kinase A (PKA) pathway (178). We wish to point out that in addition to physiological or pharmacological stimulation of cAMP production by neurohormonal secretagogues, glucose itself induces cAMP signaling in beta cells. This effect is most probably mediated by a Ca^2+^-dependent arm through Ca^2+^-dependent adenylate cyclase isoforms and by a Ca^2+^-independent arm through direct ATP availability or since the concentration of ATP is possibly saturating for adenylate cyclases even in basal glucose, through a decrease in inhibitory AMP. Moreover, cAMP oscillates in beta cells in response to stimulation by both glucose and extracellular primary messengers with the same period as electrical activity and [Ca^2+^]_IC_. The mechanism underlying these oscillations may differ depending on the experimental protocol. As [Ca^2+^]_IC_ can influence cAMP production and degradation, oscillations in cAMP may at least partly be due to oscillations in [Ca^2+^]_IC_. This seems to be supported by the observation that oscillations in [Ca^2+^]_IC_ and cAMP are typically in phase or occur with a slight shift. Pharmacological activation of GLP-1 receptors and/or direct activation of adenylate cyclase by forskolin are unable to stimulate beta cells in low glucose but can significantly enhance glucose-stimulated Ca^2+^ oscillations (180–182). Indeed, by constructing functional connectivity networks we have recently shown that increasing cAMP levels using forskolin increases beta cell activity as well as enhances synchronicity and coordination of intercellular signaling, evident as denser and more integral networks (180, 183). While both slow and fast Ca^2+^ oscillations rely on periodic entry of Ca^2+^ from extracellular space into the beta cells through voltage-activated Ca^2+^ channels, the fast Ca^2+^ oscillations may also depend on mobilization of intracellular Ca^2+^ stores from the endoplasmic reticulum. Therefore, pharmacological agents that increase cAMP may promote the appearance of fast Ca^2+^ waves (184, 185).

Many other pharmacological substances and drug candidates for diabetes treatment can affect beta cell connectivity patterns by targeting different signaling pathways, but to the best of our knowledge, apart from the cAMP-elevating agents only a few have been investigated using network measures. In this regard, glutamate signaling *via* N-Methyl-D-Aspartate receptors (NMDARs) has been demonstrated to play an important role in beta cell function by shortening the duration of bursts of electrical activity and therefore fast [Ca^2+^]_IC_ oscillations, with NMDAR antagonists being able to prolong the duty cycle by prolonging these bursts (186, 187). More specifically, following glucose metabolism, increased ATP inhibits K_ATP_ channels and causes plasma membrane depolarization, resulting in activation of voltage-dependent Ca^2+^ channels, increase in [Ca^2+^]_IC_, and insulin secretion. A negative regulation of this pathway is mediated by NMDARs. Under physiological conditions, these receptors are probably fully saturated with glutamate originating from glutamate in blood, from glutamate secreted by alpha cells, or from glutamate exported by beta cells through excitatory amino acid transporters. Membrane depolarization is therefore able to activate NMDARs, which in turn activate K_ATP_ channels and Ca^2+^- dependent K^+^ channels (SK4 channels) through a not fully understood functional interaction, thus hyperpolarizing the membrane, terminating the bursts of membrane depolarization and shortening Ca^2+^ oscillations. NMDAR inhibition interferes with this negative feedback loop and prolongs bursts and Ca^2+^ oscillations (69, 186–188). Representing the spreading [Ca^2+^]_IC_ waves as network layers, we have recently shown that the inhibition of NMDARs not only increases beta cell activity but also stabilizes and synchronizes intercellular connectivity patterns. Within consecutive [Ca^2+^]_IC_ waves, NMDAR inhibition also stabilizes the course of [Ca^2+^]_IC_ waves and the role of wave initiators (69). Given the importance of connectivity for normal and pathological functioning of islets and the fact that the network analyses are able to detect effects of non-pharmacological and pharmacological interventions beyond the classical physiological measures of beta cell activity, they are a promising tool to detect early changes during progression to diabetes and non-classical effects of new therapeutic approaches (188).

### Human Islets and Coordinated Beta Cell Activity in Health and Disease

**Figure 8:**
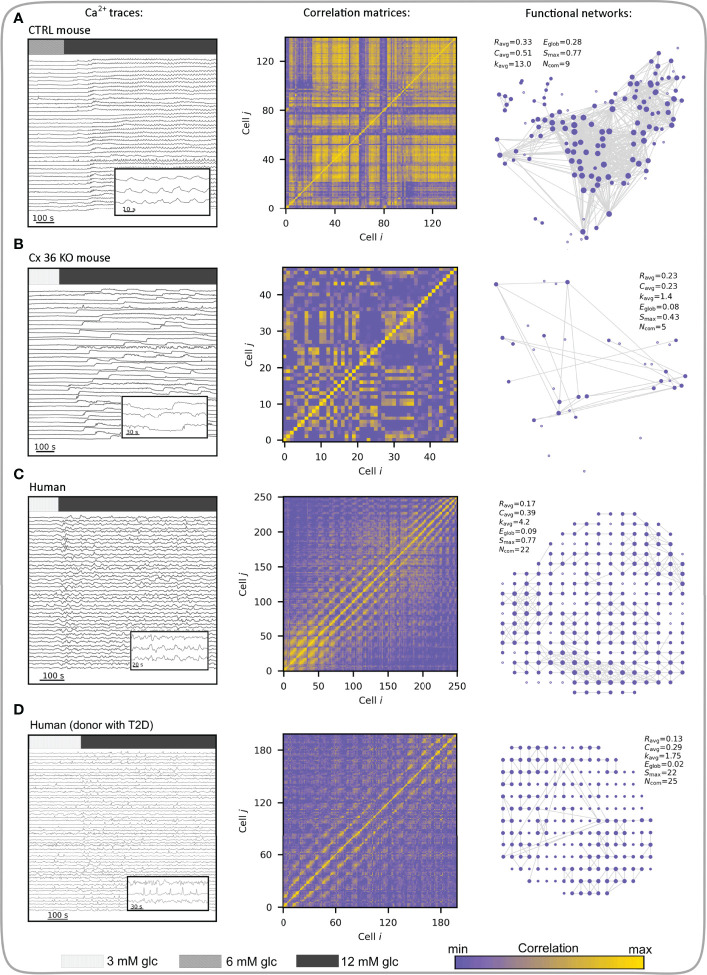
Multicellular activity and functional connectivity maps in a control (wild type) mouse islet **(A)**, an uncoupled (Cx36 KO) mouse islet **(B)**, human islet from a normal donor **(C)**, and human islet from a donor with T2D **(D)**. Left column visualizes Ca^2+^ activity with indicated stimulation intervals. Bars indicate 3- (light gray), 6- (gray) and 12- (dim gray) mM glucose stimulation. Note that 3 mM glucose was used as the substimulatory concentration in (B-D), as 6 mM can already evoke activity in these islets (unlike in control mouse islets). Inserts show short intervals of selected cellular signals. Middle column features the correlation matrices extracted from Ca^2+^ signals. Correlation levels between cell pairs are color-coded as indicated with the color bar (the same scale refers to all four panels). In the right column the corresponding functional networks are presented, which were obtained by thresholding the correlation matrices (*R*_th_ = 0.7). Along with networks, the key parameters are provided: average correlation (*R*_avg_), average clustering coefficient (*C*_avg_), average network node degree (*k*_avg_), global efficiency (*E*_glob_), largest connected component (*S*_max_), and the number of communities (*N*_com_).

During the response to glucose, human beta cells have been described to display a more regional coordination between cell clusters compared with responses in mice (189, 190), which could reflect differences in islet architecture and gap junction coupling among beta cells in different species (191–193), but at least partly also due to differences in donor age and health, mode of preparation, as well culture duration and conditions (191, 192, 194–197). In human islets, diabetes results in disrupted cytoarchitecture with altered homotypic and heterotypic communication between different cell types within an islet (105, 198). The coordinated responses of islets to stimulation with glucose, glucagon-like peptide 1 (GLP1), and glucose-dependent insulinotropic polypeptide are also altered possibly due to a reduction in Cx36 expression. Moreover, perturbed beta-cell coupling and dysregulation of Cx36-dependent [Ca^2+^]_IC_ signaling were observed in islets from donors with a high BMI, suggesting that lipotoxicity may result in lowered insulin secretion and progression to diabetes (54). Furthermore, during ageing, which is associated with increased risk of diabetes, a significant decline was found in gap junctional coupling. This was accompanied by reduced overall coordination of [Ca^2+^]_IC_ activity, which became restricted to islet subregions, and diminished insulin secretion. However, activating gap junctional communication using modafinil, a pharmacologic activator of Cx36 electrical coupling, successfully reversed the age-related decline in synchronization of [Ca^2+^]_IC_ signals (190). Supporting these observations, advanced optogenetic methods in combination with complex computational tools, confirmed differences in the coordinated responses to glucose between mouse and human beta cells. In human islets, [Ca^2+^]_IC_ waves seem to be initiated from specific subregions called pacemaker or leader regions with specific metabolic profile and local excitability (54, 64, 71, 165). The synchronization patterns are more clustered compared to mice and the collective behavior of beta cells becomes altered in diabetes (67, 145). Our own research indicates that in type 2 diabetic islets glucose-dependence is still present, but beta cell activity seems to be reduced, mostly due to a reduced oscillation frequency. Furthermore, human beta cell networks are more segregated than mouse networks and in diabetic islets more than in islets from control donors. This is accompanied by smaller and more locally restricted [Ca^2+^]_IC_ waves. Importantly, the hub regions seem to lose a disproportionately large fraction of connections, especially the long-range ones (58). **Figure 8** summarizes how differences in structural connectivity between control and Cx36 KO islets, as well as between mouse and human islets from normal and diabetic donors, translate to differences in functional connectivity. Loss of gap junction connectivity in Cx36 KO mouse islets desynchronizes beta cell [Ca^2+^]_IC_ responses, resulting in poor cell-cell correlation and sparse functional networks (**Figure 8B**). The changes in intercellular coupling are reflected in key network parameters, e.g., a lower average correlation (*R*_avg_), scarcely connected beta cells, and an overall decrease in local connectedness, as well as in global efficiency compared to the control islet (**Figures 8A, B**). Conversely, despite having a comparable average correlation as Cx36 KO islets, human islets have better connected beta cells, are more locally integrated, and have a significantly higher number of communities. Their global integration, reflected in *E*_glob_, however, is comparable to Cx36 KO mice islets, indicating a lack of long-range functional connections between subregions of the islet due to the relative lack of global [Ca^2+^]_IC_ waves compared with mice (**Figure 8C**). Clusters of beta cells in human islets form communities (while Cx36 KO islets do not), these different communities are also connected, but much less than in islets in control mice, where global [Ca^2+^]_IC_ waves and thus globally synchronized [Ca^2+^]_IC_ oscillations are the rule rather than the exception. In human islets, [Ca^2+^]_IC_ waves are heterogeneous in size and mostly encompass only smaller regions of the islet, in contrast to global, islet-wide [Ca^2+^]_IC_ waves in mice (58, 147). Both can be at least partly contributed to structural differences, particularly a higher proportion of alpha cells and a more lobular structure in human islets, promoting a higher number of heterologous contacts between alpha and beta cells and possibly introducing bottlenecks for spreading of global [Ca^2+^]_IC_ waves between the more segregated subregions (189, 192, 193). We wish to point out that despite this larger degree of segregation, human islets still seem to be able to produce [Ca^2+^]_IC_ waves that involve the great majority of islet beta cells but do so much less often compared with mouse islets where globally synchronized oscillations are the rule rather than an exception (58, 197). Due to the abovementioned structural and functional differences, human islets may even be more prone to losing the ability to produce globally synchronized [Ca^2+^]_IC_ with decreases in intercellular coupling under pathological conditions. Along this line, the functional connectivity patterns in islets from diabetic human donors (**Figure 8D**) are indeed much sparser and more segregated compared with islets from healthy controls, indicating thereby a higher fraction of inactive regions and a lack of larger and coherent [Ca^2+^]_IC_ waves (58). Finally, while much of the evidence on the role played by decreased intercellular coupling in development of diabetes presented in the last two chapters may be circumstantial, we wish to point out that when taken together, some excellent recent studies nevertheless suggest that decreased Cx36 coupling is at least partly causative. More specifically, knockout of Cx36 leads to a diabetic phenotype (78), some degree of decrease in Cx36 is present in diabetic islets and improving this by pharmacological (178, 190) or dietary interventions (178) is sufficient to improve islet function. However, more studies in this direction are needed to quantify the contribution of decreased coupling and determine whether improving coupling is a viable treatment option for diabetes.

### Computational Models of Beta Cell Networks

The new findings by network science about the complex nature of intercellular activity patterns and the specific roles played by certain cells were recognized as theoretically very appealing and have inspired the development of multicellular beta cell models. Mathematical modelling approaches have a long tradition in islet research and have also served as a paradigmatic case study for emergent networks [for excellent reviews see (60, 125, 199–201)], but the interest for the design of multicellular models has increased over the last few years. This is in part due to growing computational resources, which facilitate multiscale simulations of beta cell populations, but also due to advances in experimental techniques, which have provided valuable new data on microarchitecture and intercellular dynamics. Several studies have utilized computational models to investigate how intercellular coupling facilitates beta cell synchronization (138, 202–206) and the propagation of [Ca^2+^]_IC_ waves (74, 207, 208). In recent years, specific attention has been given to cellular heterogeneity and to how the collective activity emerges from functionally heterogeneous beta cell subpopulations (71, 143, 145, 147, 148, 165, 169, 209). In the context of complex beta cell networks, it has been demonstrated that the coupling scheme between the beta cells represents the basis for the functional connectivity patterns, but the relation relies heavily on various physiological determinants, such as the level of stimulation, cellular noise, and the coupling parameters (210). Multicellular models have also been employed to study the occurrence of long-range connections in functional beta cell networks. Importantly, it has been demonstrated that long-range synchronicity can in principle be established solely by the propagation of excitation waves through nearest-neighbor-coupled networks, if the cells and the coupling strengths are heterogeneous (142, 143, 211). In recent years, particular emphasis has also been given to the role of functional subpopulations, such as hubs and pacemaker cells, although their exact roles remain somewhat debatable. This is in part also due to the inconsistent use of terminology in the literature, but with some more recent papers this aspect is improving (55, 59, 60). It has been shown by numerical simulations that the inclusion of specialized hub cells importantly affects the collective activity on different temporal scales (212). Furthermore, by silencing a few hub cells within the islet it was possible in principle to abolish whole-islet [Ca^2+^]_IC_ activity in simulations, similarly to what was noted experimentally, but as mentioned before, only close to the threshold glucose stimulation (148). Later studies incorporating in-depth numerical analyses alleviated these findings a bit by showing that removing the metabolically most active and heavily connected cells or cells with the highest intrinsic frequencies does not diminish whole-islet activity (60, 143, 170). These results suggest that the whole-islet [Ca^2+^]_IC_ activity is probably not driven by a very small (<10%) subpopulation of extraordinary cells and that the islets are robust to loss of small groups of cells, even if they have special attributes, as would be expected for evolutionary robust assemblies of intrinsic oscillators (60, 213). To sum up, the recent theoretical works have partly confirmed some of the recent experimental findings and thereby contributed to our understanding of the complex signaling mechanisms within the islets. However, there is still a long road to drive before we will fully understand how functionally heterogeneous beta cell populations interact among each other, with other cells, and with the dynamical environment by means which may involve paracrine and other modes of intra-islet signaling (214–216), the effect of incretins (217), as well as long feedback loops, for instance between the liver and the pancreas (137). We firmly believe that the tools developed in the field of computational physiology and network science will help us to address these issues and will provide us further insights into functional properties, as well as the underlying mechanisms that guide the multicellular dynamics of endocrine cells.

## Frontiers of Islet Network Science: Assessing Multicellular Activity by Multilayer Networks and Going Beyond Calcium

### The Many Layers of Multicellular Networks

**Figure 9:**
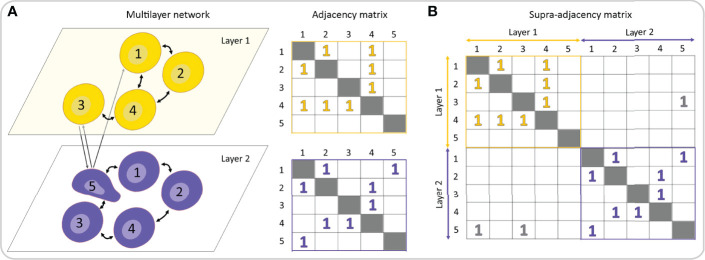
Multilayer representation of a multicellular network. **(A)** A multilayer network consists of different network layers, each one represented by an adjacency matrix. **(B)** A rank-2 tensor, generally known as the supra-adjacency matrix, can be used to describe and analyze both intra- and inter-layer connectivity.

Pancreatic islets are non-stationary complex systems governed by different oscillatory subsystems, they are characterized by different types of interactions, and their function can be captured with different measures and parameters. Therefore, the standard network approach focusing on single networks in isolation might be insufficient to unveil the functional regulatory patterns originating from complex interactions across multiple layers of physiological relationships and processes. In the last few years, the multilayer network (MLN) formalism has emerged as a new research direction to engage with such multi-dimensional systems (19, 218–221), including in the area of biomedical research (18, 222–224). By means of the MLN formalism, it is possible to track the evolution of interactions among entities over prolonged periods of time, evaluate precisely the changes caused by altered experimental conditions, such as addition of pharmacological substances or the development of a disease, explore the associations between different temporal and spatial scales, and characterize different types of interactions. In this way, a much more precise insight into the architecture and dynamics of biological systems can be acquired, compared to single-layer analyses only. While standard networks can be represented by adjacency matrices (see **Figures 4**, **8**), for MLNs higher-order matrices, i.e., tensors, are required. Formally, to represent connectivity within and between network layers, a supra-adjacency with a block structure matrix is used, in which diagonal blocks encode intra-layer connectivity and off-diagonal blocks encode inter-layer connectivity (**Figures 9A, B**). This framework allows for expansion of the traditional network analysis by examining interlayer similarity, overlapping (weighted) degrees and other measures, detection of modular super-units, identification of most central units, etc. (225, 226). In the context of beta cell networks, these MLN metrics can be used for example to assess the spatio-temporal beta cell network persistency, to quantify the effects of pharmacological interventions or to identify signal-specific functional subpopulations, as well as to determine how they change with time, as specified in more detail below.

**Figure 10:**
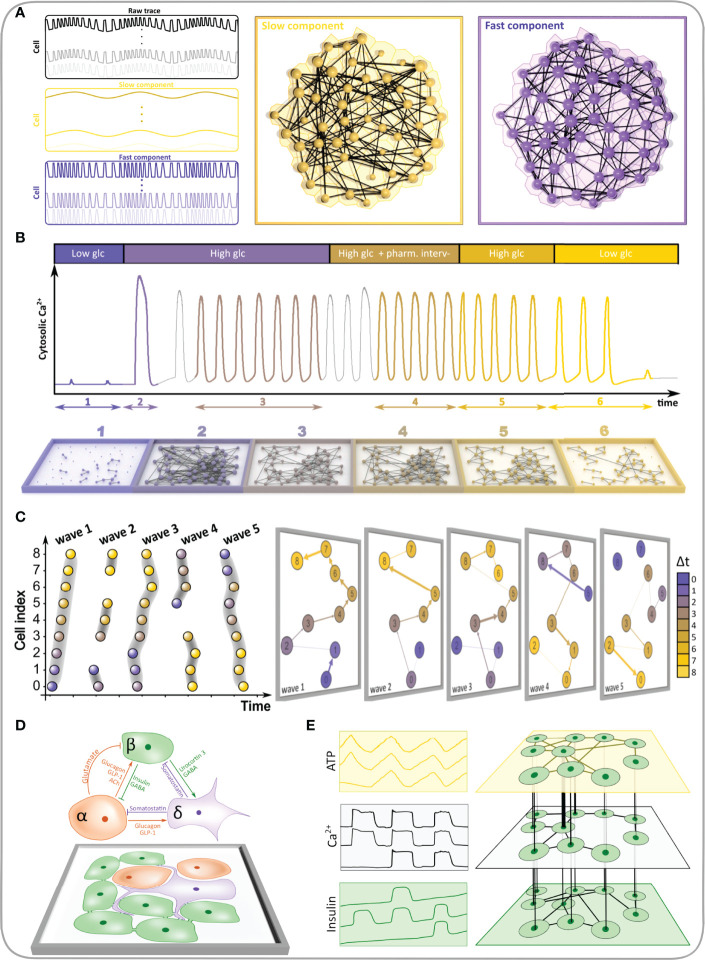
The MLN formalism can be used to study various aspects of collective dynamics and cellular activity patterns within the pancreatic islets. **(A)** A hypothetical raw [Ca^2+^]_IC_ signal and the extracted slow and fast components (left panel). The two different oscillatory components are then used to construct a two-layered multiplex functional beta cell network (right panel). **(B)** Typical [Ca^2+^]_IC_ activity in a pancreatic islet subjected to a hypothetical protocol (upper panel) and the corresponding temporal functional network layers extracted from specific time intervals (lower panel). **(C)** A schematic presentation of how network layers are designed from individual intercellular [Ca^2+^]_IC_ waves. The left panel features a raster plot indicating the onsets of oscillations of cells within specific waves and the right panel visualizes the corresponding network layers. The direction and weights of connections within each layer denote the course and the temporal lag between subsequent oscillation onsets, respectively. **(D)** Heterologous and homologous interactions within the islets visualized as a multilayer network. **(E)** Hypothetical simultaneously measured dynamics of [Ca^2+^]_IC_, intracellular ATP, and insulin release along with the corresponding functional network layers. Intralayer connections represent functional connections between time series of the same variables, whereas the interlayer connections stand for associations between different measured variables.

To date, biomedical endeavors employing the MLN concepts have been mostly limited to molecular and brain networks. In the former, the MLN formalism is predominantly used to assess the interdependent biochemical networks extracted from linked genomic, proteomic, and metabolomic data (227–230), whereas in the latter, these methods are used to address the temporal evolution of brain networks and rewiring dynamics (231–234), associations among different frequency bands (235–238), and to explore the longstanding issue about the interplay between brain structure and dynamics (36, 239, 240). On the level of tissues and intercellular interactions, the MLN methodological directions are still rather unexplored, even though the number of potential applications is large, particularly in pancreatic islets. We therefore present here some specific examples on how the MLN theory has been and could be further integrated into islet research (**Figure 10**). They are explained in continuation and encompass the assessment of different temporal scales of oscillatory activity, tracking the network evolution during prolonged stimulations or pharmacological interventions, following the course of the intercellular signals, characterization of heterologous interactions between different cell types, and the evaluation of the multicellular beta cell function by simultaneously acquiring multiple measured variables.

### Islets as Multilayer Networks

As explained above, mouse beta cells in isolated islets and in pancreatic tissue slices oscillate at different temporal scales when exposed to stimulatory glucose concentrations (56, 130, 241, 242). The slow oscillatory component with a period of several minutes is believed to reflect metabolic activity and drives the oscillatory ATP production (243, 244). Superimposed on the slow and sometimes occurring without them are so-called fast [Ca^2+^]_IC_ oscillations with a glucose-dependent frequency of around 5 min^-1^ and duration of around 2-15 s (55, 131, 159, 160), which reflect the bursting pattern of electrical activity. Both slow and fast oscillations are synchronized between different beta cells of the same islet and contribute to proper secretion patterns (133, 137, 140, 141, 245). To quantify how the multimodal oscillatory pattern manifests itself on the multicellular level, a multiplex network representation can be used (56). More specifically, using a band-pass filter, the slow and fast oscillatory component can be extracted separately from the [Ca^2+^]_IC_ recordings, and these signals can be used to construct individual network layers, as demonstrated in **Figure 10A**. Previous analyses have shown that the slow oscillations are more global, resulting in several long-range connections and networks extracted from them have a more cohesive structure compared to the networks based on fast oscillations. Moreover, there was only a weak relation between the fast and slow network layer characteristics, which suggests that different synchronization mechanisms shape the collective cellular activity in islets (56). Of note, a conceptually similar approach is commonly applied in neuroscience to construct frequency band-specific multi-layered functional brain networks (236, 237). Within this new framework, each region of the brain is mapped into a network node and replicated across all layers encoding frequency bands. Such representation of functional connectivity is better able to distinguish between brain functional connectivity patterns in health and disease (246).

The second conceivable frontier of network analyses is the investigation of network dynamics. The so-called temporal networks offer mathematically principled models of evolving networks. as well as a battery of statistical variables to characterize their evolution (19, 218). Layers are to that purpose typically generated on the basis of a series of (possibly overlapping) time windows and the nodes are linked only across sequential replicas to indicate identity. Mapping the temporal changes in connectivity patterns is meaningful in biomedical sciences as well (231, 247, 248) and could be very beneficial for the description of information flow and dynamic interaction patterns within the islets. More specifically, the activity patterns in these mini-organs are remarkably complex already under constant stimulation (147), and even more in a dynamic *in vivo* environment (67, 249, 250). In this vein, the multilayer model for time-varying networks could be used to explore fluctuations in functional connectivity during prolonged or variable stimulation of cells, as well as to assess the functional adaptation and plasticity after repeated stimulations (48, 63). Moreover, from the viewpoint of the recent developments in the field, MLN could represent a viable approach to track the network evolution after targeting specific cells *via* optogenetic and photopharmacological strategies (59, 64). In addition, with this approach one could track the changes in islets during diet-induced pathogenesis by monitoring the activity in islets transplanted into the anterior chamber of the eye (67, 249). In **Figure 10B**, a hypothetical scenario is presented, illustrating how the average temporal activity and the corresponding functional connectivity maps evolve when islets are subjected to stimulation by candidate drugs.

Third, [Ca^2+^]_IC_ waves are the main synchronizing mechanism between beta cells and their course depends heavily on cellular heterogeneity and intercellular interaction patterns. Because of the rich information encoded in individual waves, they represent the perfect candidates for being studied *via* MLN analysis. By this means, each individual wave can be regarded as an individual layer in which the connections are weighted and directed. We wish to point out that this is in stark contrast with the traditional way of constructing networks based on longer time series that include many waves and are based on similarity measures. The directionality of connections indicates the path of the intercellular signal, and the weights reflect the temporal delay between cell pairs that are subsequently activated along the course (69). We schematically present this approach in **Figure 10C**. On the left panel the activation sequence of 9 cells in 5 waves is shown in the form of a raster plot, and on the right panel the corresponding directed and weighted networks of waves are shown in their temporal order. Notably, we leveraged this approach in a recent study where we quantified the differences in collective beta cell activity between prolonged glucose stimulation only and stimulation by glucose and the NMDA receptor inhibitor MK-801 (69), which was previously shown to increase beta cell activity and synchronicity (186, 187). In our study, we focused on the velocity of intercellular [Ca^2+^]_IC_ waves and their temporal stability. Since each layer encodes the exact path of the wave (direction of connections) and the time delays between cells (weights of connections), one can compute the exact wave propagation velocity based on the geodesic path length between wave initiator cells and the last cells that activated as the sum of the weights of this path. Our analysis revealed that the propagation velocity did not change either under prolonged stimulation with glucose or under the action of NMDA receptor inhibitor. However, when quantifying the inter-layer similarity, we found that the wave initiator regions and the stability of wave paths increased dramatically when the NMDA receptors were inhibited. In this manner, we were able to identify the key factor underlying the more synchronous behavior. As such, the proposed methodology can help quantitatively evaluate the impact of pharmacological interventions on multicellular dynamics beyond classical physiological or network parameters and is applicable to other secretagogues and multicellular systems as well.

Fourth, the pancreatic islets are multicellular micro-organs that comprise predominantly alpha, beta, delta cells and communication among them is essential for proper function (73, 214, 251, 252). While direct electrical coupling through gap-junctions is the key determinant ensuring synchronous beta cell activity (76, 77) and was also found to be present between beta and delta cells (253, 254), islet cells also communicate by paracrine, autocrine, and other juxtacrine signaling pathways (73, 215, 252). It is well known that this variety of intercellular signal mechanisms is essential for a synergistic cooperation between islet cells and this represents one of the most vibrant issues in islet cell biology. With the recent advances in multicellular imaging of islet networks, our ability to dissect individual components of cell-cell communication has much improved (73). In this regard, the MLN formalism could be advantageous by providing the means to decipher the complexity of islet communication. In **Figure 10D** we illustrate a MLN representation of the endocrine cell crosstalk within the islets with some of their paracrine interactions. Within the framework of the MLN paradigm, networks of different types of cells could be constructed based on their [Ca^2+^]_IC_ signals and compared with networks based on the physical positions of cells and their paracrine or other interactions. Additionally, the relative contributions of different local signals could be dissected using the MLN approach through application of different agonists and antagonists as described in the second paragraph above. Interestingly, in a similar context, the MLN approaches have already been used to study the neural network of *C. elegans* by using multiple layers to represent different types of connections between neurons, which has led to novel findings about the functional organization of this famous neuronal network (255, 256). Very recently, muscle cells and further modes of interactions between cells have been incorporated as additional layers to this scheme, providing thereby an even deeper knowledge of the *C. elegans* connectome (257). Along similar lines, Virkar et al. proposed a multilayer network to study diffusive transport of metabolic resources among the glial network and to the synapses in the neuronal network layer (258).

Finally, as illustrated at length above, intracellular [Ca^2+^]_IC_ responses of islet beta cells represent an accessible and well-characterized approach to assess islet activity, with tremendous insight to be gained about islet behavior. However, stimulus-secretion coupling involves several steps from receptor binding or metabolism of a secretagogue to membrane depolarization and secretion of insulin granules (133, 259–261). These intracellular signaling steps interact with each other and are finely tuned by additional physiological, pathological, and pharmacological triggering and amplifying factors (133, 141, 262, 263). The [Ca^2+^]_IC_ signal itself is determined by a number of upstream processes that include glucose entry, cellular and mitochondrial metabolism, and electrical responses leading to action potential-dependent Ca^2+^ entry and an interplay with intracellular buffering and storage mechanisms (216, 261, 264). It is important to note that Ca^2+^ is not just an indicator of cellular activity but is indeed a key mediator for downstream processes required for insulin secretion itself (265), where a glucose-stimulated uptake of Ca^2+^ (266) triggers the fusion of insulin granules with the cell plasma membrane to elicit insulin release by exocytosis (267–269). Thus, while Ca^2+^ is generally a good indicator of islet cell activity, it is not reporting all relevant cell physiological processes, and indeed may not always be directly concordant with insulin secretory function. As one example, glucose stimulation can stimulate insulin secretion even when [Ca^2+^]_IC_ is clamped (270). Moreover, in (pre)diabetes increased efficacy of Ca^2+^ on the secretory apparatus can directly enhance insulin secretion (174, 271). Thus, to what extent multi-modal profiling of islet network activity should be undertaken to better understand islet behavior and dysfunction in disease? The most obvious methodological approaches to provide additional layers of data would involve simultaneous imaging of physiological processes either up- or down-stream of [Ca^2+^]_IC_ dynamics. Although the candidates described below are certainly not an exhaustive list, several measures may be considered when adding additional layers of information to these networks. More specifically, individual steps of the stimulus-secretion coupling cascade could form individual layers of MLNs and the interactions between the steps would constitute inter-layer connectivity. To date, several candidates for individual layers meet the criterion of being experimentally measurable and having an adequate spatial and temporal resolution, as well as ranging from the most proximal to the most distal steps. We wish to point out that genetically encoded sensors (including for Ca^2+^) may allow greater specificity in measurements when targeted to the cell cytosol, mitochondria, or other relevant intracellular organelles or sub-cellular locations, and greater cell specificity when expressed under the control of relevant promotors (for example the insulin promoter) in engineered model animals, such as mice or zebrafish, or when delivered to human islet cells, typically by adenoviral vectors (181).

If we follow the stimulus-secretion coupling cascade, although cellular glucose uptake can be measured using a fluorescent probe (272), and this can be combined with [Ca^2+^]_IC_ responses in islet cells (273), additional information on the metabolic activity of islet cells can also be gained using approaches that more directly assess relevant metabolic signals. In this regard, sensors reporting glycolysis (the most proximal signaling step) have provided insight into the regulation of oscillatory metabolic activity and can be characterized by oscillations in phosphofructokinase-1 (PFK1) activity and its product fructose-1,6-bisphosphate (FBP) (274), and pyruvate kinase activity (244, 275). Moreover, several approaches to monitor mitochondrial or cellular metabolic function have been adopted for use in pancreatic islets over the last decades. NAD(P)H autofluorescence is a long-used indicator of islet cell metabolic function (276–278), the signal which is generally dominated by mitochondrial activity (279). Chemical probes reporting mitochondrial membrane potential have been used to monitor mitochondrial activity within islets coincident with [Ca^2+^]_IC_ responses (244, 280, 281). Additionally, mitochondrial oscillations can be optically monitored by measuring the signals from mitochondrial flavins (244). Furthermore, sensors for NADPH (282) or H_2_O_2_ (283) have provided insight into redox-dependent control of islet function. Genetically encoded probes for ATP have revealed signaling microdomains (284) and the relationship between mitochondrial Ca^2+^ and cytosolic ATP/ADP ratios (285). Combined imaging of these and other relevant metabolic signals and mitochondrial function (including lactate, glutamate, and mitochondrial pH) have recently provided important insight into the metabolic control of insulin secretion and prompted a reconsideration of the consensus model for stimulus-secretion coupling (286). New and improved genetically-encoded probes for key signaling molecules, for example recent new probes for citrate (287) and lactate (288), may also provide for improved imaging to allow collection of additional layers of data relating metabolic and [Ca^2+^]_IC_ networks in islets.

Although the abovementioned approaches can all be combined with Ca^2+^ signaling networks, it is worth noting that other relevant signals can be assessed. Imaging of islet cell membrane potential has been accomplished using small molecule voltage-sensitive probes (289), and may perhaps be more robustly measured using newer genetically encoded voltage indicators (290). Moreover, optogenetic control of islet function is also feasible in transgenic models (291). Further, as already mentioned, signaling by G-protein coupled receptors *via* second messengers, such as cAMP, is important in both physiology and diabetes treatment (292), and such second messengers are even important for the maintenance of beta-cell activity at a baseline level (293). Genetically encoded probes for cAMP demonstrate glucose-dependent cAMP responses (182) and intra-islet signaling (294). Finally, approaches to monitor the downstream process of insulin secretion from islet cells of intact islets typically involve the visualization of extracellular probes at a cellular resolution. This can include the visualization of zinc ions (Zn^2+^) released into the extracellular space concomitantly with insulin (295). Indeed, many Zn^2+^-binding dyes suitable for this purpose are available (296). Markers of the extracellular volume, such as sulphorhodamine B, have also been used to monitor insulin secretion from individual cells within islets (297), including evaluation of the spatiotemporal control of individual insulin exocytosis event (298). Thus, in the context of intact islet cellular network activity as measured by [Ca^2+^]_IC_ responses, abundant opportunity exists to add additional layers assessing upstream (metabolism, cAMP), coincident (voltage responses), and downstream (exocytosis) physiological processes. **Figure 10E** shows an example of an MLN that comprises oscillations in [Ca^2+^]_IC_ (upper panel), out-of-phase oscillations in [ATP]_IC_ (middle panel), and in-phase oscillations in secreted insulin (lower panel). Along the same line, the MLN approach has already been used to elucidate the relationship between functional connectivity patterns based on membrane potential and [Ca^2+^]_IC_ signals (299). In perspective, such approach may be for instance instrumental in dissecting the properties of first responders, wave initiators, hubs, and other cells in terms of their biochemical properties, their sensitivity to glucose, and their secretory potential. In other words, MLNs may help address the question whether a cell that seems to be important in one layer also has any special roles in other layers.

Network-based analyses of islets of Langerhans are typically studied in islets isolated from the pancreas and therefore lacking in the extracellular matrix, vascularization, and innervation that play important roles in signal transduction (300). However, other models may allow the observation and construction of islet cell networks during development, after transplantation into a live animal, and in the context of disease. The tissue slice approach can be regarded as a first in the series of possible upgrades to conditions that are closer to the *in vivo* situation (301–303). Further, the zebrafish *Danio rerio* is transparent during development and thus has islet tissue that can be readily visualized (304). By introducing stable fluorescent probes, zebrafish can therefore be a model for studying glucose-stimulated [Ca^2+^]_IC_ dynamics in islet cells within the local microenvironment. A similarly transparent site is the anterior chamber of the eye of rodents, where islets can be transplanted, become highly vascularized, and [Ca^2+^]_IC_ dynamics can be visualized (249). This site can be used to study not only human or mouse islet networks in an environment closer to the native pancreas, but also the characteristics of networks and cell-cell signaling in developing stem cell-derived islet-like clusters (305). Moreover, this approach can be employed in the context of a model for type 1 diabetes, wherein beta cells are attacked by the immune system (306), and in the context of type 2 diabetes using high fat diet or other rodent models (271), or donor islets from people who lived with the disease (249). Combining and comparing network properties from isolated islets and islets analyzed *in situ* will continue to offer insights into islet communication, both in non-diabetic and diabetic states.

## Conclusion

Since the end of the 20^th^ century, complex networks have become a common and irreplaceable language in interdisciplinary studies and over the last two decades, the field of network science has experienced an explosive growth (98, 307). The research interests have evolved in several directions, from social and economic systems (3, 308, 309), to a wide range of engineered and technological systems (86, 87, 310), and to the more recently developing field of multilayered networks (19, 219). Guided by the advances in high-throughput data-collection and imaging techniques, network analyses are also becoming an indispensable tool in biomedical sciences across multiple disciplines and levels of organization (15, 36, 82, 85), including in studies of intercellular interactions in tissues (18, 59). Understanding how heterogeneous populations of interconnected cells in a dynamic and noisy environment operate to ensure proper function is very appealing and challenging to investigate. In the present contribution, we focused on how the network approaches have been and can be used to study the pancreatic islets of Langerhans. Work in this field has stimulated research collaborations across different disciplines, from experimental to advanced modelling and computational approaches (58–60, 124, 141, 242, 263). For such collaborations to be even more fruitful in the future, experimentalists need to have a good basic understanding of network science and network scientists need to have a detailed understanding of islet biology. In our view, this is today even more important than it was at the birth of islet networks a decade ago, since the number of experimental and analytical options is increasing at an unprecedented pace. Our wish for the present review is to serve as an information hub for islet biologists and network scientists, helping them navigate through the complex network of existing knowledge and future options and finding the way to each other.

## Author Contributions

All authors listed have made a substantial, direct, and intellectual contribution to the work, and approved it for publication.

## Funding

The work presented in this study was financially supported by the Slovenian Research Agency (research core funding Nos. P3-0396, P1-0055 and I0-0029, as well as research projects Nos. J3-3077, J1-2457, J3-9289, N3-0048, N3-0170, and N3-0133). PM holds the Canada Research Chair in Islet Biology.

## Conflict of Interest

The authors declare that the research was conducted in the absence of any commercial or financial relationships that could be construed as a potential conflict of interest.

## Publisher’s Note

All claims expressed in this article are solely those of the authors and do not necessarily represent those of their affiliated organizations, or those of the publisher, the editors and the reviewers. Any product that may be evaluated in this article, or claim that may be made by its manufacturer, is not guaranteed or endorsed by the publisher.

## Footnotes

1 In the case of points with only even numbers of lines attached to them, it does not matter where the walk starts. In the case with two points with odd numbers of lines attached to them, the walk must start in one of them.
